# Cerebrovascular Resistance: The Basis of Cerebrovascular Reactivity

**DOI:** 10.3389/fnins.2018.00409

**Published:** 2018-06-19

**Authors:** James Duffin, Olivia Sobczyk, Larissa McKetton, Adrian Crawley, Julien Poublanc, Lashmi Venkatraghavan, Kevin Sam, W. Alan Mutch, David Mikulis, Joseph A. Fisher

**Affiliations:** ^1^Department of Physiology, University of Toronto, Toronto, ON, Canada; ^2^Department of Anaesthesia and Pain Management, University Health Network, University of Toronto, Toronto, ON, Canada; ^3^Institute of Medical Science, University of Toronto, Toronto, ON, Canada; ^4^Joint Department of Medical Imaging and the Functional Neuroimaging Lab, University Health Network, Toronto, ON, Canada; ^5^Department of Anesthesia and Perioperative Medicine, University of Manitoba, Winnipeg, MB, Canada

**Keywords:** cerebrovascular reactivity, carbon dioxide, magnetic resonance imaging, cerebrovascular resistance, model, humans

## Abstract

The cerebral vascular network regulates blood flow distribution by adjusting vessel diameters, and consequently resistance to flow, in response to metabolic demands (neurovascular coupling) and changes in perfusion pressure (autoregulation). Deliberate changes in carbon dioxide (CO_2_) partial pressure may be used to challenge this regulation and assess its performance since CO_2_ also acts to change vessel diameter. Cerebrovascular reactivity (CVR), the ratio of cerebral blood flow (CBF) response to CO_2_ stimulus is currently used as a performance metric. However, the ability of CVR to reflect the responsiveness of a particular vascular region is confounded by that region’s inclusion in the cerebral vascular network, where all regions respond to the global CO_2_ stimulus. Consequently, local CBF responses reflect not only changes in the local vascular resistance but also the effect of changes in local perfusion pressure resulting from redistribution of flow within the network. As a result, the CBF responses to CO_2_ take on various non-linear patterns that are not well-described by straight lines. We propose a method using a simple model to convert these CBF response patterns to the pattern of resistance responses that underlie them. The model, which has been used previously to explain the steal phenomenon, consists of two vascular branches in parallel fed by a major artery with a fixed resistance unchanging with CO_2_. One branch has a reference resistance with a sigmoidal response to CO_2_, representative of a voxel with a robust response. The other branch has a CBF equal to the measured CBF response to CO_2_ of any voxel under examination. Using the model to calculate resistance response patterns of the examined branch showed sigmoidal patterns of resistance response, regardless of the measured CBF response patterns. The sigmoid parameters of the resistance response pattern of examined voxels may be mapped to their anatomical location. We show an example for a healthy subject and for a patient with steno-occlusive disease to illustrate. We suggest that these maps provide physiological insight into the regulation of CBF distribution.

## Introduction

Cerebral blood flow (CBF) is distributed via an extensive vascular network, where flow to a particular region, is allocated according to a number of regulatory mechanisms that operate via vascular smooth muscle to change vessel diameter and hence the resistance to flow. Cerebrovascular resistance also responds to carbon dioxide (CO_2_), and cerebrovascular reactivity (CVR), the change in blood flow in response to alterations in CO_2_, is used as a metric to test cerebrovascular regulatory ability (Lythgoe et al., 1999; Vesely et al., 2001). However, the ability of CVR to reflect the responsiveness of a particular vascular region is confounded by that region’s inclusion in the cerebral vascular network, where all regions respond to the global CO_2_ stimulus. Consequently, local CBF responses reflect not only changes in the local vascular resistance but also changes in local perfusion pressure resulting from redistribution of flow within the network. As a result, the CBF responses to CO_2_ take on various non-linear patterns that are not well-described by straight lines (Fisher et al., 2017). Thus, local changes in flow, do not necessarily reflect local changes in resistance, so that the latter cannot be inferred from the former.

Our aim is to distinguish the CVR status of a region in terms of a physiological parameter, cerebrovascular resistance. We propose using a simple model to convert measured CBF responses to CO_2_ to the resistance responses that underlie them. We suggest that anatomical mapping of the resistance response pattern sigmoid parameters provides physiological insight into the regulation of CBF distribution. We note that this presentation is limited to a description of the development of the model, including its limitations, a parameter sensitivity analysis, and a brief description of its application. Although we believe that this model analysis will prove useful, a demonstration of its value must await further experience.

### Anatomy and Physiology Background

**FIGURE 1:**
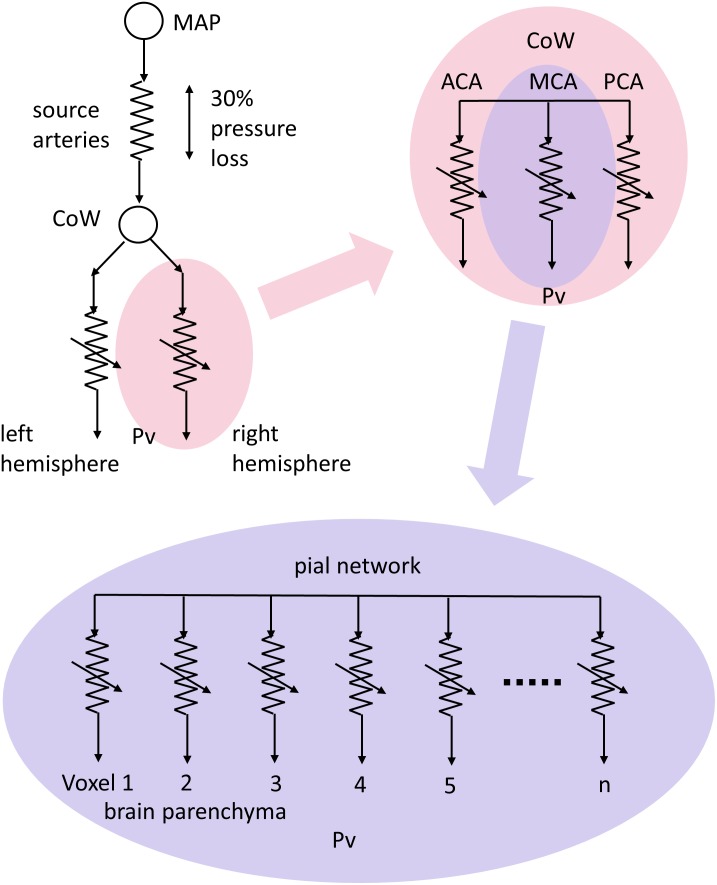
A conceptual schematic of cerebral blood flow (CBF) pathways and resistances. Resistances are represented with electrical resistance symbols with variability indicated by an arrow. Mean arterial pressure (MAP) forces flow through the high resistance of the source arteries to anastomose around the circle of Willis (CoW) where they are distributed to vascular territories, via the anterior, middle and posterior cerebral arteries (ACA, MCA, and PCA, respectively) in each hemisphere. The interconnected pial networks supply the brain parenchyma via penetrating arterioles, with flow finally reaching the tissue volumes seen in magnetic resonance imaging (MRI) scans through regulating resistances to venous pressure (Pv).

The blood supply to the brain features several unique anatomical and physiological characteristics that mitigate against a loss of supply (Willie et al., 2014). Anatomically, the circle of Willis acts to redistribute blood flow upon abrupt failure of a systemic supply artery (Zarrinkoob et al., 2015), and long-term compensation involves the development of collateral pial and intracerebral vessels (Moody et al., 1990; Liebeskind, 2003). A simplified schematic of the blood flow pathway to a small vascular bed, such as that within a voxel measured with magnetic resonance imaging (MRI), can be considered as pictured in **Figure 1**. Blood flows from the aorta via the major extra-cerebral source arteries (e.g., carotid arteries) to a distribution network that is highly redundant due to multiple parallel pathways (e.g., interconnections within the circle of Willis and pial vasculature), then to the voxel vascular bed via smooth muscle-lined vessels.

Physiologically, CBF is largely controlled via vascular smooth muscle. Multiple layers of vascular smooth muscle cells cover the large pial arteries on the surface of the cortex, which branch into penetrating arterioles sheathed in a single layer of vascular smooth muscle cells (Nishimura et al., 2007), and enter the cortical parenchyma where capillary control may occur (Hall et al., 2014; Attwell et al., 2016). Two mechanisms control vascular smooth muscle cells to regulate regional blood flow by manipulating vascular diameter and hence vascular resistance. (i) Autoregulation maintains resting blood flow despite variations in brain perfusion pressure (Tan and Taylor, 2014; Tzeng and Ainslie, 2014), and (ii) neurovascular coupling increases local blood flow in response to increased metabolic demand (Attwell et al., 2011, 2016; Phillips et al., 2016). All of these factors act in concert to change vascular resistance by altering vascular diameter according to circumstances.

### The CBF Response to CO_2_

Cerebrovascular resistance also responds to CO_2_, and, in health, the balanced changes in regional vascular resistances in response to a progressive global increase in CO_2_ results in a symmetrical, stereotypical sigmoidal pattern of progressive increase in blood flow in most regions of the brain (Battisti-Charbonney et al., 2011; Bhogal et al., 2014; Sobczyk et al., 2014), as long as arterial blood pressure remains constant (Regan et al., 2014). However, in patients with localized cerebral vascular disease resulting in single or multifocal regions of vascular stenosis, due to for example atherosclerosis or vasculitis, the changes in blood flow with a progressive hypercapnic stimulus become asymmetrical. Healthier vessels draw a disproportionate flow of blood at the expense of the more compromised vessels, and as a result the flow response patterns deviate from a sigmoidal response pattern. Indeed, in a survey using a ramp of a controlled, increasing CO_2_ stimulus to explore the full range of cerebrovascular responses, we found that the blood-oxygen-level dependency (BOLD) responses in voxels measured with MRI take on any of four patterns. These may be described as increasing, decreasing, inverted U-shaped and U-shaped (Fisher et al., 2017), with only the increasing pattern (the normal response) as a sigmoidal pattern. With such non-linear responses a more sophisticated method of analysis is needed than a linear fit of BOLD vs. CO_2_.

### A Conceptual Model for CO_2_-Induced Redistribution of CBF

**FIGURE 2:**
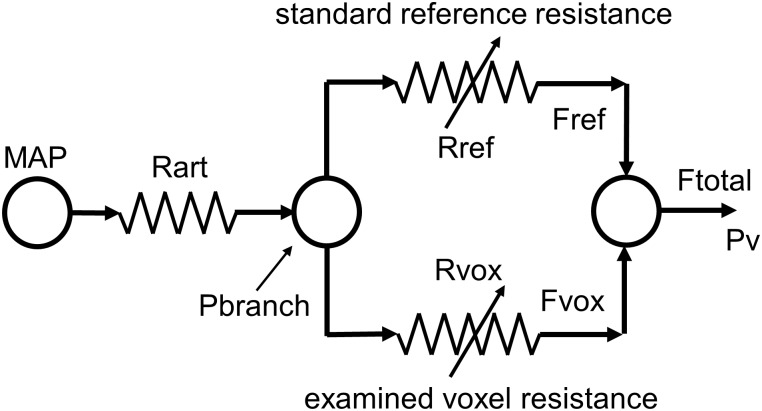
The resistance model; determining the pattern of resistance change from the pattern of blood flow change during a ramp CO_2_ stimulus. MAP, mean arterial blood pressure; Pv, venous blood pressure; Rart, major arteries resistance; Pbranch, perfusion pressure at branch; Rref, standard reference branch resistance; Fref, standard reference branch blood flow; Rvox, examined voxel branch resistance; Fvox, examined voxel branch blood flow; Ftotal, total blood flow through Rart.

To fit the different patterns of BOLD response to CO_2_ we used an approach based on the conceptual model shown in **Figure 2**. This model takes into account the significant resistance of the major blood vessels (Rart) that conduct blood flow to the brain (Faraci and Heistad, 1990; Warnert et al., 2016), and can explain the paradoxical reduction in flow observed during a vasodilating stimulus (Sobczyk et al., 2014) first described as cerebrovascular steal (Brawley, 1968; Symon, 1969; Conklin et al., 2010) or a reverse Robin Hood phenomenon (Alexandrov et al., 2007).

A global CO_2_ stimulus causes vasodilation in both branches, and consequently a decrease in branch pressure because of the upstream resistance of the major cerebral arteries. If an unhealthy branch cannot vasodilate, and the other healthy branch can reduce its resistance to increase its flow, a reduction in branch pressure results and consequently the flow in the unhealthy branch decreases and steal results. It should be noted that the venous pressure is assumed to be zero in this model. This simplified model therefore not only explains the steal phenomenon but also shows that any mismatch of resistance responses between the two branches will alter the distribution of flow between them. Thus, CBF is distributed to brain regions such that flow is preferentially routed to regions of lowest resistance.

Using this resistance model of the interactions between the two branches it is possible to calculate the patterns of the resistance responses to a ramp CO_2_ stimulus of each voxel from their BOLD response patterns (Duffin et al., 2017). These resistance responses to CO_2_ are sigmoidal, no matter what the BOLD-CO_2_ response pattern, because of limitations to vasodilation and vasoconstriction at high and low end-tidal partial pressures of CO_2_ (PETCO_2_) respectively. The simplified model provides a way of fitting the various BOLD patterns of response to CO_2_ that has a physiological basis; an interaction among the network of vascular resistances whose responses to CO_2_ have a sigmoidal pattern.

### A Standardized Resistance Response Pattern

This application of the model can be further developed. As previously described (Duffin et al., 2017) its use was limited to voxel BOLD response observations in individual subjects, choosing pairs of BOLD patterns of response to CO_2_ for the model vascular beds, one from a voxel with a robust response serving as a standard comparator for any other voxel BOLD response pattern. This use of a robust BOLD response pattern as a standard comparator for other voxel BOLD response patterns in an individual subject led to the idea of using a resistance sigmoid response pattern as a standard comparator instead. A standard resistance sigmoid pattern can thus be used in one vascular bed of the model as the comparator for BOLD response patterns observed in any voxel.

With such a standard comparator, the BOLD response patterns in any voxel can be examined to determine the model resistance pattern of response for that voxel. That pattern is sigmoidal, and since it has been derived for each voxel with the model using the same standard comparator resistance response pattern, all voxel resistance pattern sigmoid parameters can be compared between voxels in that subject and individualized resistance parameter maps generated. An extension of the standard comparator concept is to use the same standard reference resistance response pattern for interrogating the resistance of any voxel in any subject. Anatomical maps of the voxel resistance sigmoidal response parameters allow comparisons based on anatomical location within and between subjects, analogous to the CVR analysis described by Sobczyk et al. (2015).

## Methods

### Experimental Protocol

#### Subjects

The example data used to illustrate the resistance maps was drawn from our database of healthy control subjects and patients with cerebrovascular disease that had undergone a standardized testing protocol as a result of participation in a Research Ethics Board (REB) approved study at our institution. The data used here was from 38 (25F) control subjects aged 18–76, mean (SD) = 41.6 (16.7), and 10 patients with clinical symptoms and/or known cerebrovascular disease, (4F) aged 23–72, mean (SD) = 45.4 (16.5). Each control subject was in good health, a non-smoker, not taking medication, and had no history of cardiovascular, respiratory or cerebrovascular disease, had no structural lesions on their anatomical scan, and did not have hypertension or diabetes. The patients were chosen to reflect a wide variety of cerebrovascular dysfunction. These studies conformed to the standards set by the latest revision of the Declaration of Helsinki and were approved by the REB of the University Health Network and Health Canada. All subjects in the database were competent and gave written informed consent.

#### Data Acquisition

**FIGURE 3:**
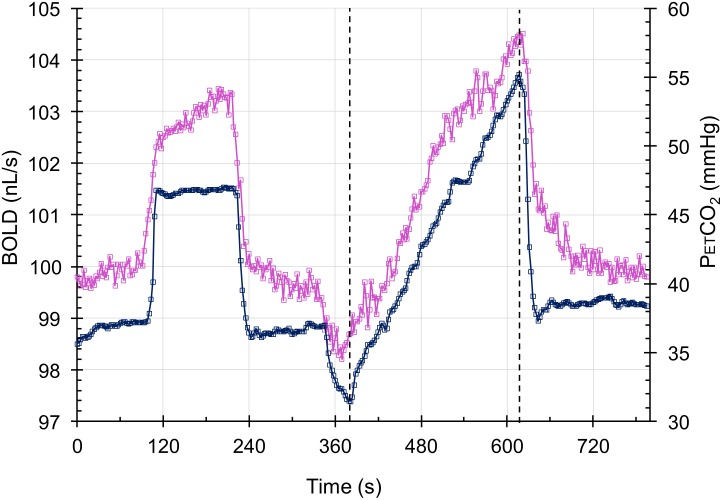
The test protocol. The CO_2_ stimulus (blue squares) and the whole brain average BOLD response scaled to 100 nL/s at resting PETCO_2_ (magenta squares) in a control subject. The step change is used for measuring the speed of response in a separate analysis, and the ramp portion shown between the dashed lines is used in assessing the resistance response.

During testing, subjects breathe via a face mask, connected to a sequential gas delivery breathing circuit (Somogyi et al., 2005). Targeting PETCO_2_ was via sequential gas delivery (Slessarev et al., 2007; Fisher et al., 2016) implemented by a computerized gas blender (RespirAct, Thornhill Research, Inc., Toronto, ON, Canada). The CO_2_ stimulus sequence is illustrated in **Figure 3** and consists of clamping PETCO_2_ at the subject’s resting baseline for 2 min, a step increase of 10 mmHg for 2 min followed by 2 min at baseline. PETCO_2_ is then slowly reduced by hyperventilation over 30 s, followed by a steady rise in PETCO_2_ at a rate of 0.1 mmHg/s to 15 mmHg or more above baseline over 4.0 min, and a return to baseline for 2 min. With this CO_2_ targeting approach PETCO_2_ has been shown to be equivalent to the arterial partial pressure of CO_2_ (PaCO_2_) (Ito et al., 2008; Willie et al., 2012).

The BOLD acquisitions with echo planar imaging gradient echo (TR/TE = 2400/30 ms, 3.5 mm isotropic voxels, field of view 24 cm × 24 cm, 39 slices, slice thickness 3.5 mm, matrix size 64 × 64, number of frames = 405, flip angle = 70°) were obtained using a 3.0-Tesla Signa HDx scanner with an 8-channel phased-array receiver coil (GE Healthcare, Milwaukee, WI, United States). The acquired MRI and PETCO_2_ data were analyzed using AFNI software (Cox, 1996). BOLD images were then volume registered and slice-time corrected and co-registered to an axial 3-D T1-weighted Inversion-Recovery prepared Fast Spoiled Gradient-Echo (IR-FSPGR) volume (TI/TR/TE = 450/8/3 ms, matrix size 256 × 256, field of view 22 cm × 22 cm, slice thickness = 1 mm, and flip angle = 15°) that was acquired during the same scan session (Saad et al., 2009).

#### Data Analysis

PETCO_2_ data was re-sampled at the TR of the BOLD scan, and time-shifted to the point of coincidence with the mean brain BOLD signal where PETCO_2_ abruptly decreased at the end of the ramp. The time shift is done to time align the BOLD and PETCO_2_ data that were recorded on different computers. The amount of shift therefore has no physiological significance as the data acquisitions were not synchronized.

Cerebrovascular reactivity was calculated from the slope of a linear least-squares fit of the BOLD signal data series to the PETCO_2_ data series over the range of PETCO_2_ represented by the ramp portion of the CO_2_ sequence on a voxel-by-voxel basis. The CVR was color-coded to a spectrum of colors corresponding to the direction (positive or negative) and the magnitude of the slope.

### Resistance Model Description

The resistance model pictured in **Figure 2** is labeled with the variables used in the analysis. Note that the model constants and variables of pressure, flow and resistance although given their respective units, they do not correspond with actual measurements in the brain. Pressure units were chosen as mmHg, and nL/s were arbitrarily chosen as the unit of model flow, making resistance units mmHg/nL/s and the appropriate axes in the graphs are labeled accordingly. The following assumptions apply:

1. Mean arterial pressure (MAP) and Rart are constants (MAP is arbitrarily set to 100 mmHg and Pv to zero).
2. Pbranch, Rref, Fref, Rvox, Fvox, and Ftotal are variables that change with PETCO_2_.
3. Rref changes sigmoidally with PETCO_2_, with fixed parameters a, b, c and d according to the following Eq. 1:

(1) $$\text{Rref}=\text{a}+\text{b}/(1+\exp(-{\text{PETCO}}_{2}-\text{c})/\text{d}))$$

Where:

Rref is resistance (pressure/flow, mmHg/nL/s), a function of PETCO_2_

(a) is the maximum resistance in hypocapnia (mmHg/nL/s);
(b) is the sigmoid amplitude = minimum–maximum resistance (mmHg/nL/s);
(c) is the sigmoid midpoint PETCO_2_ where slope (sensitivity) is maximum (mmHg);
(d) is the PETCO_2_ range over which resistance is linear (mmHg).

The model uses the pattern of the BOLD response to PETCO_2_ as the pattern of the Fvox response to PETCO_2_. To scale between the measured BOLD and the model flow, the BOLD signal at the resting PETCO_2_ (BOLDrest) was arbitrarily set to 100 nL/s, and its pattern of response to CO_2_ is calculated from the change in BOLD with PETCO_2_ from that at resting PETCO_2_ as described in Eq. 2.

(2) Fvox=100*BOLD/BOLDrest

Since the model is based on the pattern of BOLD response to CO_2_, which is a relative change, this convention scales the model parameters to values that are convenient. This choice and that of MAP = 100 mmHg, determine both Rart and the Rref sigmoid parameters, as well as the calculated Rvox resistance sigmoid parameters. However, although these choices change the absolute values in the model, the pattern of Rvox resistance change in response to CO_2_ remains unchanged. Thus, the values of Rart and MAP only affect the scaling of the patterns of the resistance changes with CO_2_, and alternative choices could have been used (see Supplementary File).

#### Calculating Voxel Resistance Response Patterns

Once the BOLD response is converted to Fvox, the pattern of the Rvox response to PETCO_2_, is determined by calculating Rref from Eq. 1 at each PETCO_2_ of the ramp stimulus, and then calculating Rvox at each PETCO_2_ with Eq. 3. The resulting pattern of the Rvox response to the ramp PETCO_2_ stimulus is then fitted with the sigmoid Eq. 4 using a constrained Levenburg–Marquardt least absolute residual algorithm (LabVIEW, National Instruments, Austin, TX, United States). The Rvox sigmoid parameters derived from the model can then be color coded and mapped onto anatomical maps.

(3) Rvox=(MAP−Fvox*Rart)/(Fvox*(1+Rart/Rref))

(4) $$\begin{array}{c} \text{Rvox}=\text{Start}+\text{Amplitude}/(1+\exp(-{\text{PetCO}}_{2}\\ -\text{Midpoint})/\text{Range)}) \end{array}$$

**FIGURE 4:**
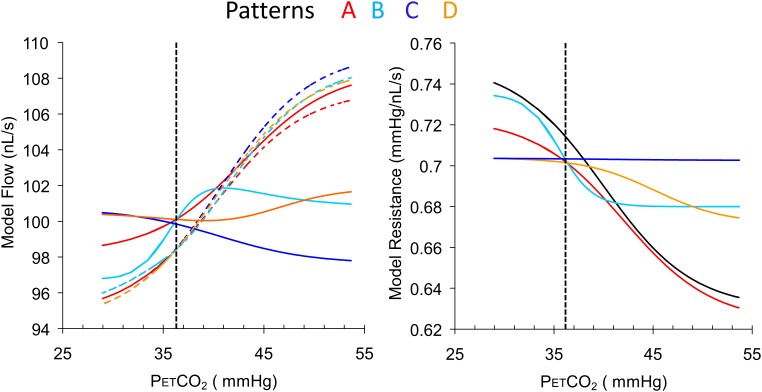
Examples of the four types of response patterns for model flow **(left)** and the calculated resistance response patterns **(right)** with the reference resistance (black line). The four voxel branch flow patterns (color coded solid lines) are increasing (A, red), inverted U-shaped (B, light blue), declining (C, dark blue) and U-shaped (D, orange), with the accompanying reference branch flow patterns shown as dashed lines. The dotted vertical line indicates the subject’s resting PETCO_2_. Note that despite the varying shapes of the BOLD curves, the resistance curves are all sigmoidal in shape as expected except type C, which is unresponsive. The choice of BOLD scaling to model flow of 100 nL/s at the resting PETCO_2_ brings the model resistances at resting PETCO_2_ to approximately the same value for all patterns.

**Figure 4** illustrates the use of the model to convert four patterns of BOLD responses to a ramp PETCO_2_ stimulus observed in a control subject (resting PETCO_2_ = 35.5 mmHg) into resistance response patterns.

### Determining the Model Constants

The constants of the model were established from a survey of the patterns of the mean BOLD response to a ramp PETCO_2_ stimulus in brain regions we judged representative of a *functionally* healthy response (CVR between 0.5 and 0.6%/mmHg) in each of 38 healthy control subjects. To find the constants we proceeded as follows:

(1) In each subject, both branch blood flows of the model, Fref and Fvox, were set equal to a scaled version of the mean healthy BOLD pattern of response to the ramp PETCO_2_ stimulus, where BOLD at resting PETCO_2_ was set equal to a model flow of 100 nL/s as previously described in Eq. (2). Thus, each branch of the model had the same flow, i.e.,

(5) Fref=Fvox

With this constraint the two resistances will be identical because they are connected in parallel, i.e.,

(6) Rref=Rvox

(2) To determine Rart, it was assumed that a 30 mmHg pressure drop occurred from MAP to Pbranch, based on the findings detailed in Faraci and Heistad (1990). Rart was calculated from the Fref and Fvox scaled values at the resting PETCO_2_ using Eq. 7 and since the flow in both branches was scaled to 100 nL/s at resting PETCO_2_, Rart becomes 30/200 = 0.15 mmHg/nL/s.

(7) Rart=30/(Fref+Fvox)

(3) Using this value of Rart, the Rref response to the ramp PETCO_2_ stimulus was determined from:

(8) Rref=(MAP−(Fvox+Fref)*Rart)/Fref

where: Fvox and Fref were given by Eqs (2) and (5).

Finally, the sigmoid parameters a, b, c, and d associated with Rref were estimated using a constrained Levenburg–Marquardt least absolute residual algorithm (LabVIEW, National Instruments, Austin, TX, United States).

### Determining the Resistance Sigmoid Fitting Constraints

To investigate CVR status in regions that may not be functionally healthy, a survey of 10 patients with various types of cerebrovascular disease was undertaken. We assumed that the model parameters for Rref remained unchanged, but Fref and Fvox were no longer the same. With Fvox given by Eq. (2), the local voxel BOLD response, and Rref given by Eq. (1), we calculated Rvox from Eq. (3). The four sigmoid parameters of Eq. (4) could then be estimated using the least absolute residual fitting algorithm for Rvox mentioned above. This analysis was used to determine the voxel resistance sigmoids for all voxels in each patient. Histograms were made for each parameter and each subject to discern the range of each parameter that would typically be encountered in a patient, and these determined the sigmoid fitting constraints.

## Results

### The Model Constants

**FIGURE 5:**
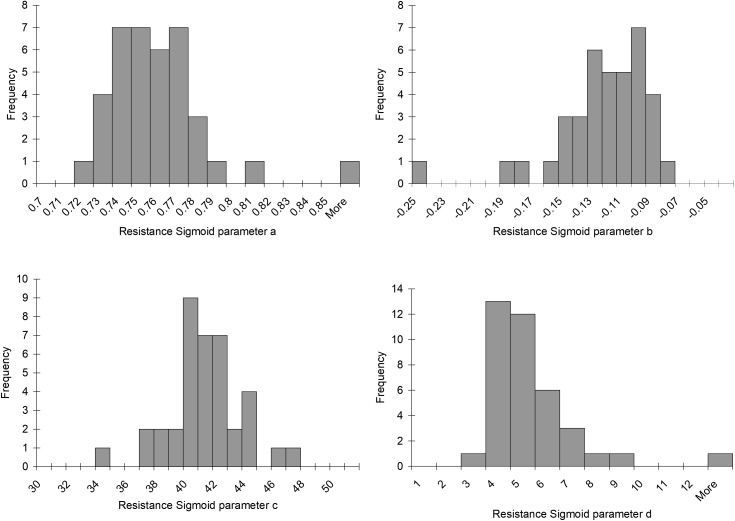
Histograms of the resistance sigmoid parameters observed in the survey of 38 healthy control subjects.

**FIGURE 6:**
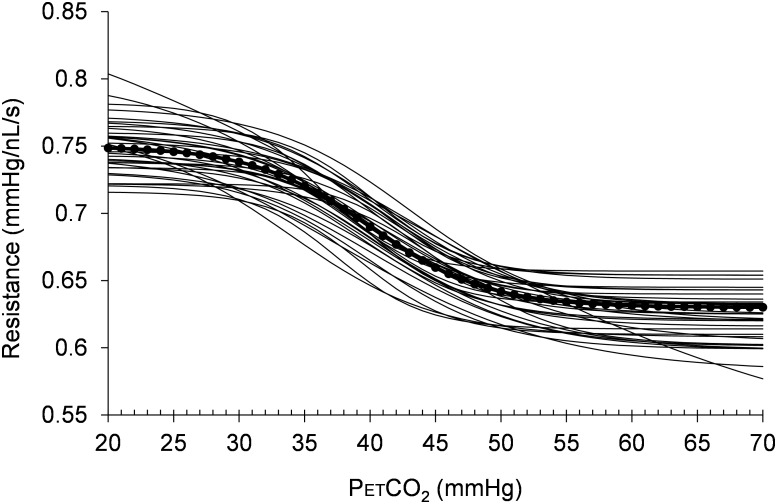
The resistance sigmoids of the healthy regions for all 38 healthy control subjects surveyed. The dotted line shows the reference sigmoid chosen for the model from the median values of the cohort sigmoid parameters.

Histograms of the values for the resistance sigmoid parameters a, b, c, and d found in the survey of the patterns of the mean BOLD response to a ramp PETCO_2_ stimulus in brain regions we judged representative of a *functionally* healthy response are presented in **Figure 5**. The Rref sigmoid parameters, were set to the median values found in the survey of 38 control subjects. **Figure 6** shows the sigmoids for all subjects in the survey and the Rref sigmoid based on the median values of the population sigmoid parameters. Rref sigmoid parameters a and b determine the reference resistance and its overall extent of change during the ramp PETCO_2_ stimulus and were set to a = 0.75 and b = -0.12, respectively. The Rref sigmoid midpoint constant c reflects the PETCO_2_ where sensitivity is a maximum, and was set to 40 mmHg, the median value found in the survey. The Rref range constant d reflects the range of PETCO_2_ over which the Rref sigmoid can be considered linear, and thus affects the shape of the sigmoid response; it was set to 4.5 mmHg, the median value we observed in our survey. These choices establish the pattern of the reference standard resistance sigmoid response to a ramp PETCO_2_ stimulus to which any other voxel resistance sigmoidal response can be compared using the model.

### The Resistance Sigmoid Fitting Constraints

**FIGURE 7:**
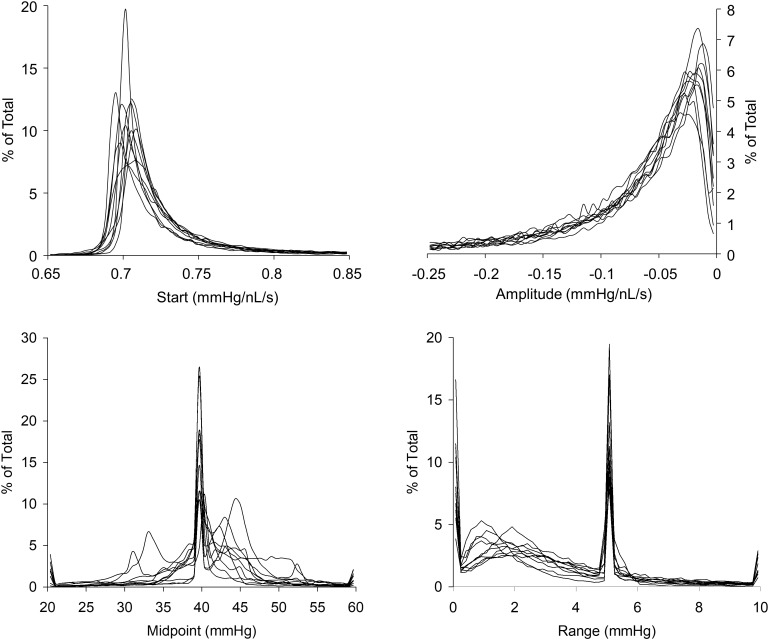
Histograms of the resistance sigmoid parameters for all voxels in the 10 patients with cerebrovascular disease.

The resistance sigmoid parameter histograms as shown in **Figure 7** show the variation in parameter values over all voxels from the survey of 10 patients with various types of cerebrovascular disease. They indicate that the most common midpoint was approximately 40 mmHg, and the most common range was approximately 5 mmHg. Since the midpoint is the PETCO_2_ at which reactivity sensitivity is maximum and the range is the span of PETCO_2_ over which reactivity to PETCO_2_ is linear, these two measures therefore indicate the common characteristics of the cerebrovascular response to CO_2_ in this group of 10 patients. As well as setting the display scale limits, the skewedness of these parameter histograms was also reflected in the map color scales for start, amplitude and midpoint deviation, whose colors were distributed in a square law fashion in order to highlight small changes for the most common values. The parameter constraints and display scale limits listed in **Table 1** were chosen based on these histograms.

**Table 1:** Constraints and scales for the resistance sigmoid parameters.

Parameters	Fitting constraints	Display scale limits
Start	0.6 to 1.0	0.65 to 0.85
Amplitude	-0.001 to -0.3	-0.001 to -0.25
Midpoint	20 to 60	20 to 60
Range	0.001 to 10	0.001 to 10

### Example Resistance Parameter Maps

**FIGURE 8:**
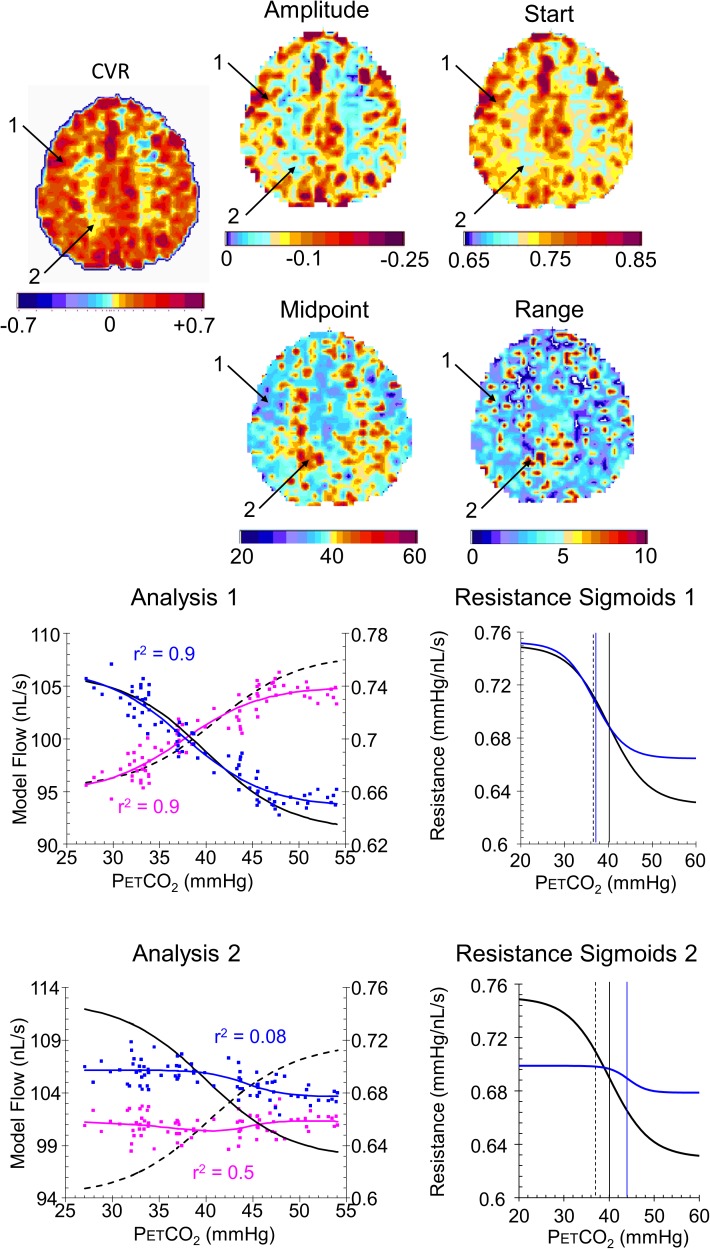
Example maps for a single axial slice and their color scales from a healthy control subject with analysis graphs and resistance sigmoids from two example voxels. The maps show the locations of example voxels 1 (high amplitude, midpoint at resting PETCO_2_) and 2 (low amplitude, high midpoint). The analysis graph shows the model fitting process, using the reference resistance (black solid line) and its calculated flow (black dashed line): model voxel branch flow pattern of response to PETCO_2_, scaled from the % changes in BOLD vs. PETCO_2_ (magenta points), is converted to resistance (blue points), then fitted with a sigmoid (blue line). The flow patterns of response for the voxel branch and reference resistance branch are then calculated from the resistance response patterns (voxel, blue line and reference black dashed line) The resistance sigmoids graphs show the relation of the fitted voxel sigmoid resistances (blue line) to the reference resistance sigmoid (black line), with their respective midpoints indicated by the vertical lines. The dashed vertical line shows the subject’s resting PETCO_2_.

**FIGURE 9:**
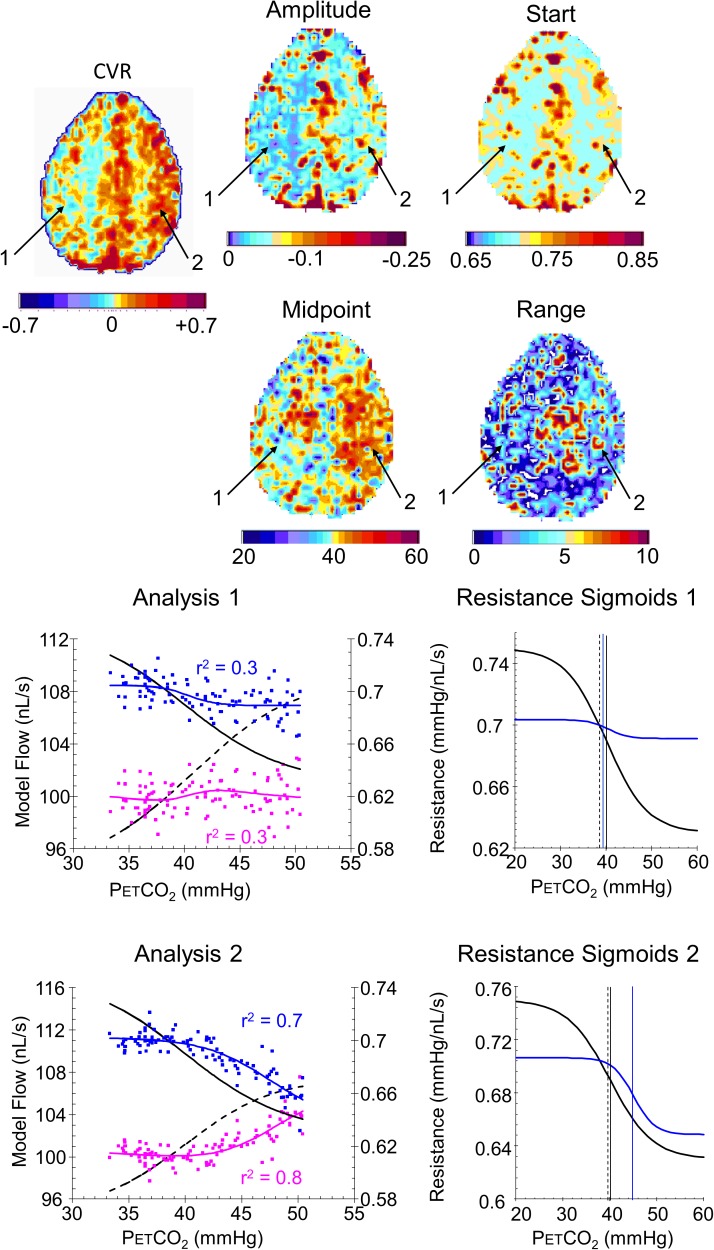
Example maps for a single axial slice and their color scales, with analysis graphs and resistance sigmoids from two example voxels for a patient with a near occlusion of the right carotid and bilateral foci of stenosis in the vertebral arteries. The maps show the locations of example voxels 1 (low amplitude, midpoint at resting PETCO_2_) and 2 (high amplitude, high midpoint). The rest of the caption is the same as for **Figure 8**.

**FIGURE 10:**
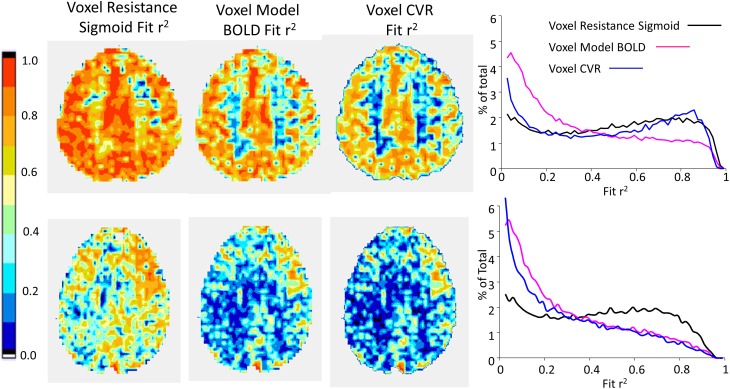
Example maps for a single axial slice of r^2^ fit assessments for the subjects shown in **Figure 8** (top row) and 9 (bottom row), with histograms of the r^2^ fits for all voxels.

**Figures 8**, **9** compare CVR maps and resistance parameter maps for a healthy control subject and a patient with steno-occlusive disease. These examples show that maps of the voxel resistance parameters describing the resistance sigmoid response to a ramp of PETCO_2_ provide further information about the physiology of blood flow distribution in the brain. **Figure 10** shows the that the model analysis provides a better fit for the BOLD pattern of response to PETCO_2_ than the single line of the CVR, and maps of these r^2^ assessments, especially for the resistance sigmoid fit, provide not only assurance that the assumption of a sigmoid was correct, but also identify regions where noisy data and little or no response occurs.

## Discussion

### General

First, we must emphasize that this model is not an anatomical one. The resistances are not actual, nor are the model flows; only the patterns of model flow and resistance changes with CO_2_ are considered, based on the relative changes in BOLD responses to CO_2_. Thus, it could be viewed as an exercise in pattern fitting, a way of fitting the variety of patterns observed in the BOLD response to CO_2_, especially in patients, that has a common physiological basis. The model is simply a way of providing a standardized method based on the physiological assumptions of network interactions affecting a voxel’s perfusion pressure and a sigmoidal change in resistance with CO_2_; the latter supported by the existence of physical limits of vasoconstriction and vasodilation and by the high r^2^ fitting values obtained over diverse regions. Given that this standardized fitting procedure does account for the variety of observed BOLD-CO_2_ response patterns, we suggest that the resistance sigmoid parameters of the examined voxels describe underlying physiological effects.

The choice of model flow scaling to BOLD and the choices of MAP and Rart do change the absolute values of resistance of the model branches but not the resistance responses in terms of the pattern of change with CO_2_. The choice of reference resistance sigmoid parameters determines the calculated parameters for the voxels, but, since they are all related to the same reference, maps of the voxel resistance sigmoid parameters are relative maps; only the color scaling changes with different reference resistance parameters. The effect of such model parameter variations are examined in a Supplementary File.

One way of viewing this model approach to the interpretation of the BOLD-CO_2_ response of a voxel is to enable a separation of the innate resistance response to a vasoactive stimulus such as CO_2_ from the confounding effects of local perfusion pressure changes resulting from the network redistribution of flow during a global vasodilatory stimulus. The resulting resistance sigmoid characterizes the ability of a region (i.e., voxel or vascular bed) to respond to local regulatory demands, such as those made by neurovascular coupling. For this reason, we suggest that the resistance parameter maps provide an insight into vascular physiology and pathophysiology not discernable from conventional CVR maps, and may therefore prove useful to understanding pathophysiological changes in disease.

Indeed, the examples shown for a control subject (**Figure 8**) and patient (**Figure 9**) identify characteristics that are not discernible from the CVR maps, such as voxel 2 in the control subject that has a low amplitude as might be expected from the CVR map but a midpoint, where the responsiveness is a maximum, not at resting PETCO_2_, but at an elevated PETCO_2_. By contrast, voxel 2 in the patient also has an elevated midpoint in a region of high CVR and amplitude. These observations require a physiological explanation and as further experience with these maps is gained valuable insights may accrue.

### Model Assumptions

The assumptions made for this model are very similar to those presented in a previous paper (Duffin et al., 2017). The model uses single variable resistances to represent the equivalent total resistances of vascular capillary beds, a conceptual simplification of the actual vascular anatomy. The major difference between the previous and present models is the adoption of a universal standard resistance response sigmoid for the reference branch here, rather than the BOLD-CO_2_ response of a healthy voxel for the individual subject under study in the previous model. This change came about after accumulating experience with the previous model analysis. We noted that the reference branch resistance responses calculated from the BOLD-CO_2_ responses of healthy voxels in many subjects had similar sigmoid parameters. Consequently, it became apparent that it would be possible to adopt a standard resistance response sigmoid that could be used in the model for all subjects, rather than one that was unique to each subject.

The standard resistance response used for the reference branch is intended to simulate the effect of the vascular network on the perfusion pressure experienced by the examined voxel branch of the model during a global stimulus. Previous studies of CBF have shown that redistribution during a global vasodilatory stimulus depends on the relative CVR; high CVR regions affect regions of low CVR (Sobczyk et al., 2014), resulting in cerebrovascular steal in extreme cases (Conklin et al., 2010). The reference branch resistance response does not produce the actual change in perfusion pressure experienced by the examined voxel branch but is a way of exposing every examined voxel branch to the same challenge, in all subjects.

During the ramp CO_2_ challenge, it is assumed that cerebral metabolism, neural activation as well as MAP and Rart are unaffected by CO_2_. Subjects are supine during these measurements and are instructed to relax but may invoke neural activation in response to their surroundings to an unknown extent. Whether hypercapnia changes cerebral metabolism is debateable (Yablonskiy, 2011), but it can affect MAP (Battisti-Charbonney et al., 2011), and if so remains a confound (Regan et al., 2014). Until MRI compatible devices capable of measuring MAP continuously, only intermittent MAP measures are available, and in our experience most subjects do not show a significant rise in MAP (>10 mmHg) during these experiments. For those subjects that do, the model results must be interpreted with considerable caution. Rart is fixed for the model but may change with very high CO_2_ since all cerebral vessel smooth muscles are relaxed by CO_2_ (Willie et al., 2014). However, the small vessels of vascular beds are the most sensitive (Wei et al., 1980) and dominate the resistance changes with CO_2_ so that the model is a reasonable description of the pattern of vascular bed resistance changes with CO_2_.

Finally, we emphasize that the success of this model analysis depends on controlling the PETCO_2_ stimulus so that a slow ramp increase occurs over a wide stimulus range from hypocapnia to hypercapnia, with the hypocapnic range requiring the cooperation of subjects to hyperventilate while the RespirAct^TM^ controls the target PETCO_2_ (i.e., prevents it from going below target) independent of ventilation. In this way the PETCO_2_ stimulus should reach the limits of vasodilation and vasoconstriction that define the sigmoidal response. It is assumed that the speed of response does not influence the relations between flow distribution and CO_2_ (Blockley et al., 2011; Duffin et al., 2015; Poublanc et al., 2015) because the ramp stimulus is sufficiently slow. If this assumption is incorrect then any very slow response regions would show an increased resistance sigmoid midpoint.

### Limitations

The first point of discussion must consider the validity of this model analysis. It should be understood that the model analysis is dependent on the assumption that every voxel examined experiences perfusion pressure changes due to the vasodilatory response of other brain regions. Should no such perfusion pressure changes occur during the CO_2_ stress, then the model premise is violated and the predicted sigmoidal resistance is incorrect. We are nevertheless confident that such competition exists (Brawley, 1968; Symon, 1969; Sobczyk et al., 2014) and accounts for the wide variety of BOLD-CO_2_ response patterns observed (Fisher et al., 2017).

The model uses a fixed sigmoidal pattern of resistance change with CO_2_ for the reference branch because the actual resistance change with CO_2_ is unknown. The resistance sigmoid parameters for all examined voxels are therefore calculated in the model from the measured BOLD-CO_2_ response using the same standard resistance response in the reference branch. In this way the calculated resistance response for the examined voxel fits the pattern of its measured BOLD response to CO_2_. Consequently, the calculated sigmoid parameters for the examined voxel resistance are not measures of the actual resistance response but relative measures. Thus, we are not able to compare voxel absolute resistances, only comparisons between the sigmoid parameters describing the pattern of resistance response to a ramp of CO_2_.

The model analysis depends on the assumptions listed in Section “Methods.” First, that brain metabolism and neural activation is unchanged during the ramp PETCO_2_ challenge. Although, this assumption has been challenged by results from steady-state experiments (Xu et al., 2011) its validity is unknown for slow changes in PETCO_2_ such as the ramp challenge we used. We suggest that the effect of brain metabolism and neural activity on the BOLD signal that occurs over the duration of the ramp stimulus is very small compared to the vascular effect of CO_2_.

One of the common criticisms of studies such as this one is concerns with the fact that BOLD is only a surrogate rather than a direct measure of CBF. These concerns have been discussed at length in previous papers (Duffin et al., 2015, 2017; Fisher et al., 2017) and the interested reader can find them there. In brief, first, although BOLD deviates from a linear relationship to CBF at very high flows (Hoge et al., 1999), the increases in BOLD we observed were <10%, which is within the linear BOLD-CBF relation according to the Davis [dHb] dilution model (Davis et al., 1998). Second, BOLD measures are affected by cerebral blood volume (CBV), which also changes with hypercapnia. However, the fractional change in CBV relative to baseline is approximated by the fractional change in CBF relative to baseline raised to the power of 0.2 (Chen and Pike, 2010; Mark and Pike, 2012), and therefore likely to have little effect on the sigmoidal responses we measured. Third, BOLD may also reflect changes in cerebral metabolic rate, and changes in oxygen tension (Bulte et al., 2007; Prisman et al., 2008). With respect to metabolic rate the assumption that hypercapnia does not change cerebral metabolic rate is debateable (Yezhuvath et al., 2009; Yablonskiy, 2011), and must therefore be regarded as a caveat for these experiments. We maintained normoxic isoxia during these tests to avoid changes in oxygen tension. Finally, we used the subject’s resting PETCO_2_ as baseline with the ramp from hypocapnia to hypercapnia passing through this PCO_2_, because the BOLD-PETCO_2_ relationship depends on the baseline PETCO_2_ (Cohen et al., 2002; Sobczyk et al., 2014; Halani et al., 2015).

At present BOLD is a widely available method that provides a sufficient spatial and temporal resolution to be useful, and methods to obtain similar resolutions with direct flow measurement methods are not widely available. Moreover, where a direct comparison of BOLD and flow measures such as arterial spin labeling (ASL) are made, the authors conclude that ASL MRI confirms that, even in patients with steno-occlusive disease, the BOLD MRI signal response to hypercapnia predominantly reflects changes in CBF (Mandell et al., 2008), and this conclusion is supported by recent experiments comparing BOLD and positive emission tomography (Fierstra et al., 2018). Therefore, we suggest that, for this initial exploration of a method to derive cerebrovascular resistance response patterns, measures of BOLD response patterns to a CO_2_ stimulus ramp are appropriate. When improvements in CBF measurement become available the method can be applied to them.

That the flow resistance of the supply artery (Rart) is fixed and does not change with CO_2_ requires investigation. Current experiments find that hypercapnia does affect the larger supply arteries (Willie et al., 2012), but are confounded by a concomitant rise in MAP (Regan et al., 2014). This assumption has the advantage of simplifying the model, but we suggest that a more complex model incorporating changes of Rart with CO_2_ may not make a significant difference to the calculated resistances because of the low CO_2_ sensitivity of the major vessels; however, this assumption remains to be tested.

## Conclusion

The development of an analysis method to generate the resistance sigmoid parameter maps presented here evolved from a simple model of interaction between two vascular beds, which was used as a way of separating the two effects on the BOLD-CO_2_ response pattern of a vascular bed; the innate resistance response to CO_2_, and the changes in perfusion pressure resulting from the redistribution of flow during a global CO_2_ stimulus. Since the response of a vascular bed to a vasoactive stimulus is not usually to a global stimulus, we suggest that the innate resistance response to a vasoactive stimulus may describe the response to a local stimulus such as the metabolic demand of neurovascular coupling. In that case, the resistance sigmoid parameter maps should provide considerable insight into the underlying physiology and pathophysiology of the control of the CBF and prove to be of clinical value. This conclusion therefore merits further testing as experience with resistance sigmoid parameter maps accumulate in patients with a wide range of neurological and cerebrovascular diseases.

## Author Contributions

OS, LM, and KS data collection. OS, LM, JP, and JD data analysis. JD, AC, WM, LV, DM, and JF concept and design. JD, WM, DM, and JF manuscript drafting. JD, OS, LM, AC, JP, LV, KS, WM, DM, and JF manuscript revision and approval.

## Conflict of Interest Statement

JF and JD are senior scientists at Thornhill Research, Inc., (TRI), a spin-off company affiliated with the University Health Network that developed the RespirAct^TM^, a non-commercial research tool assembled by TRI to enable cerebrovascular reactivity studies. DM is a shareholder in TRI. OS is a part time employee of TRI. The remaining authors declare that the research was conducted in the absence of any commercial or financial relationships that could be construed as a potential conflict of interest.
