# Supplementary material for: Cerebrovascular Resistance: The Basis of Cerebrovascular Reactivity
Source: Front Neurosci. 2018 Jun 19;12:409. doi: 10.3389/fnins.2018.00409 (PMC6020782; doi:10.3389/fnins.2018.00409)
Supplement: Supplementary file 1 [file Presentation_1.PDF]

Model parameter variations

Rart

In the model an Rart of 0.15 represents a decrease in pressure from the mean arterial pressure to the branch pressure of 30 mmHg (30%). With a decrease of 20 or 40 mmHg the corresponding Rart values are 0.1 and 0.2 respectively. These different values of Rart simply change the absolute value of the calculated model resistances, without changing the shape (i.e. parameters) of the model resistance sigmoids as shown in Figure 1A. Only the examined voxels sigmoid parameter Start map is affected and a change of scale provides the same colour map as shown by the example maps in Figure 1B

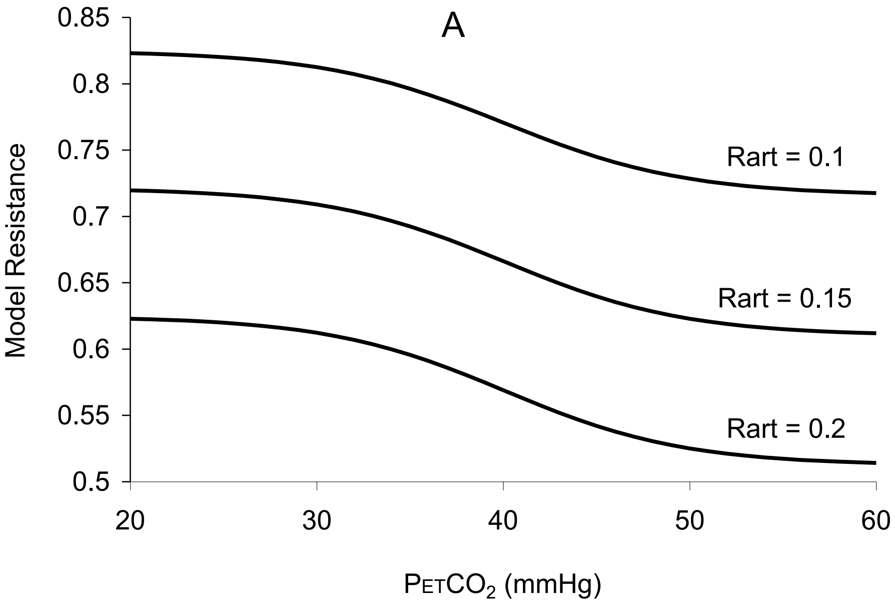

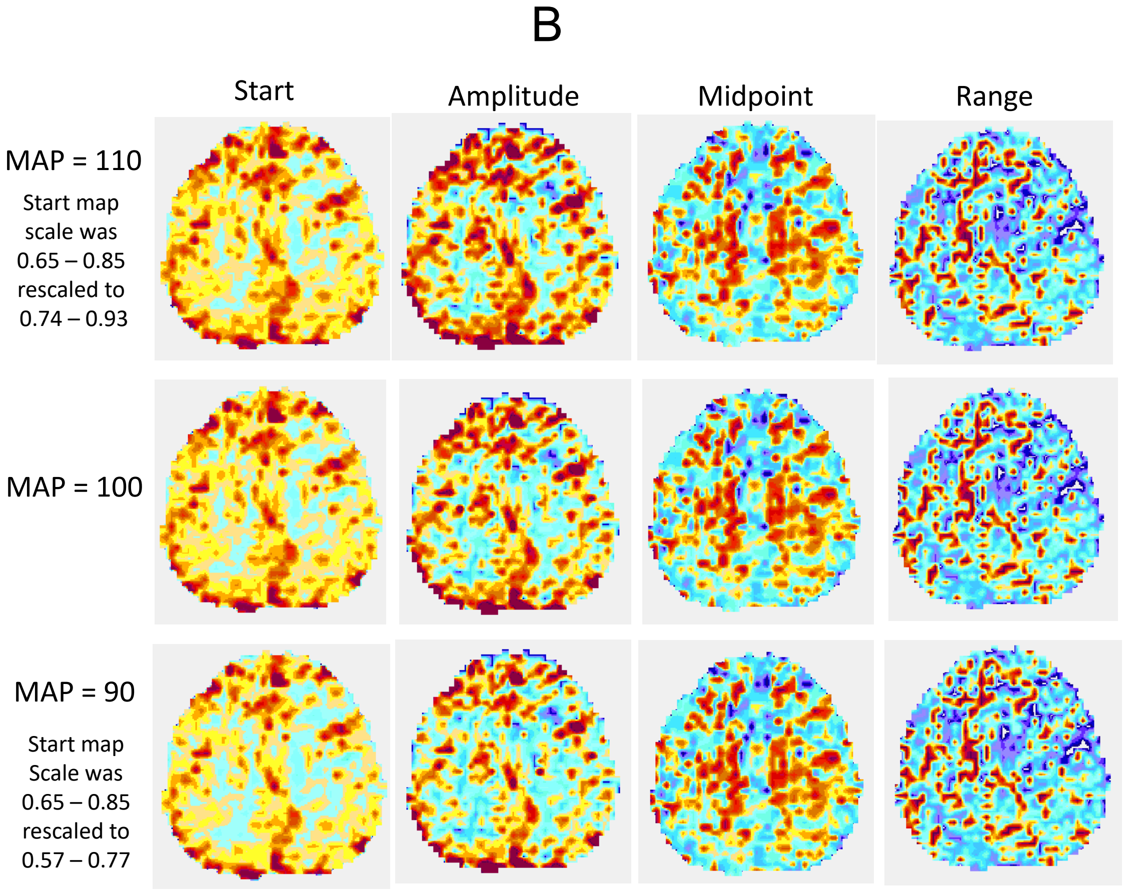

Figure 1: The effects of a change in Rart on model resistance (A) and an example of the examined voxels resistance sigmoid parameter maps (B)

MAP

Changes in MAP have a similar effect as Rart in that different values of MAP simply change the absolute value of the calculated model resistances, without changing the shape (i.e. parameters) of the model resistance sigmoids as shown in Figure 2A. Only the examined voxels sigmoid parameter Start map is affected and a change of scale provides the same colour map as shown by the example maps in Figure 2B.

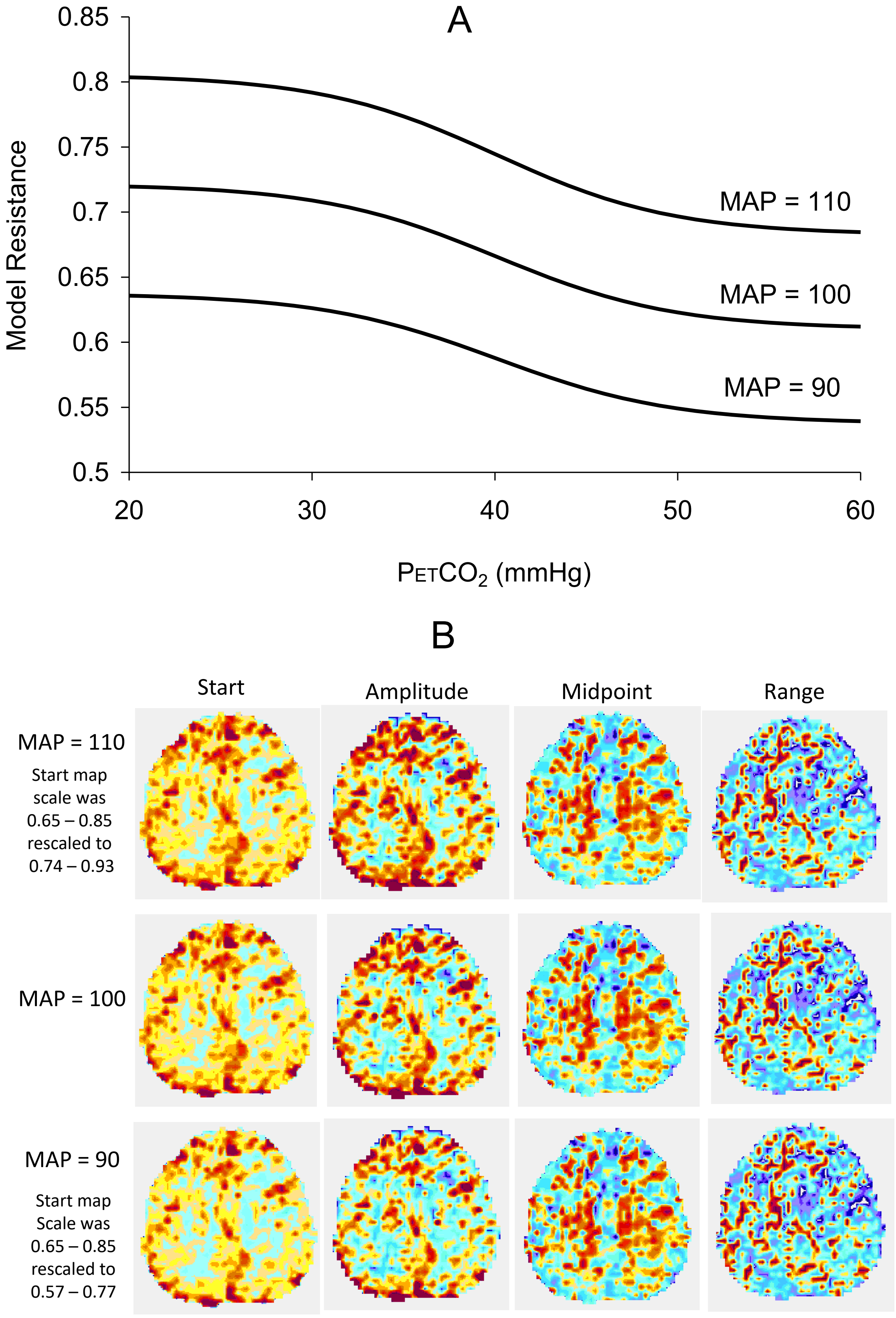
A
Model Resistance
0.85
0.8
0.75
0.7
0.65
0.6
0.55
0.5
20
30
40
50
60
MAP = 110
MAP = 100
MAP = 90
$P_{ET}CO_2$ (mmHg)
B
Start
Amplitude
Midpoint
Range
MAP = 110
Start map
scale was
0.65 – 0.85
rescaled to
0.74 – 0.93
MAP = 100
MAP = 90
Start map
Scale was
0.65 – 0.85
rescaled to
0.57 – 0.77

Figure 2: The effects of a change in MAP on model resistance (A) and an example of examined voxels resistance sigmoid parameter maps (B)

Reference resistance parameters (Start)

Reference resistance sigmoid parameter a (Start) variation changes the maximum value, without changing the shape (i.e. parameters) of the resistance sigmoids as shown in Figure 3A. Only the examined voxel Start parameter map is affected and a change of scale provides the same colour map as shown by the example maps in Figure 3B.

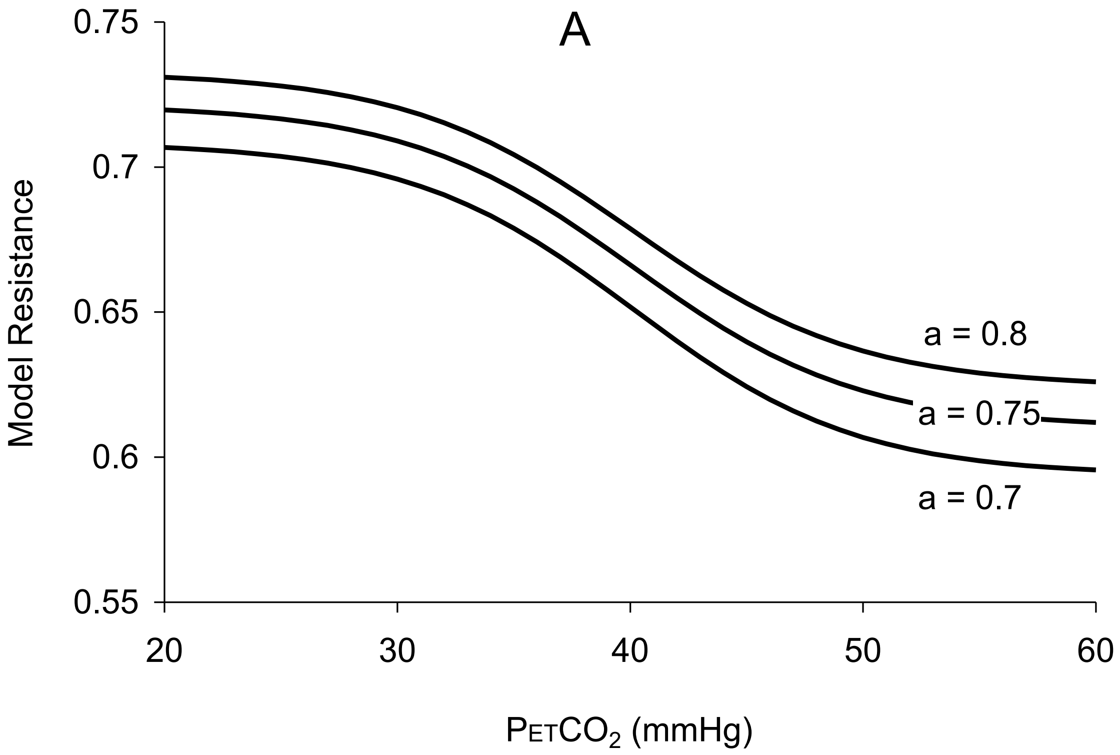

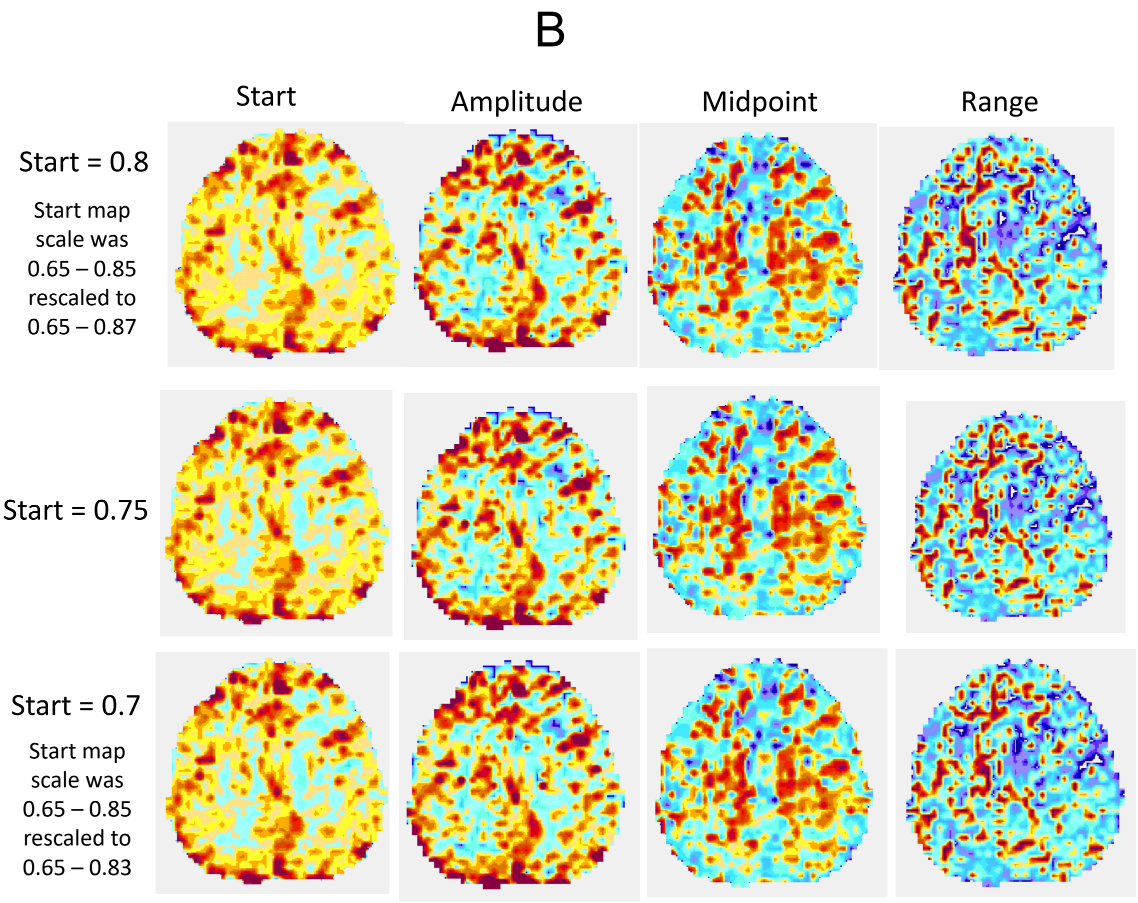

Figure 3: The effects of a change in reference resistance sigmoid parameter a (Start) on model resistance (A) and an example of the examined voxels resistance sigmoid parameter maps (B)

Reference resistance parameters (Amplitude)

Reference resistance sigmoid parameter b (Amplitude) variation changes the amplitude of the calculated examined model resistance sigmoid as shown in Figure 4A, without substantially affecting the examined voxels resistance parameter maps (Figure 4B).

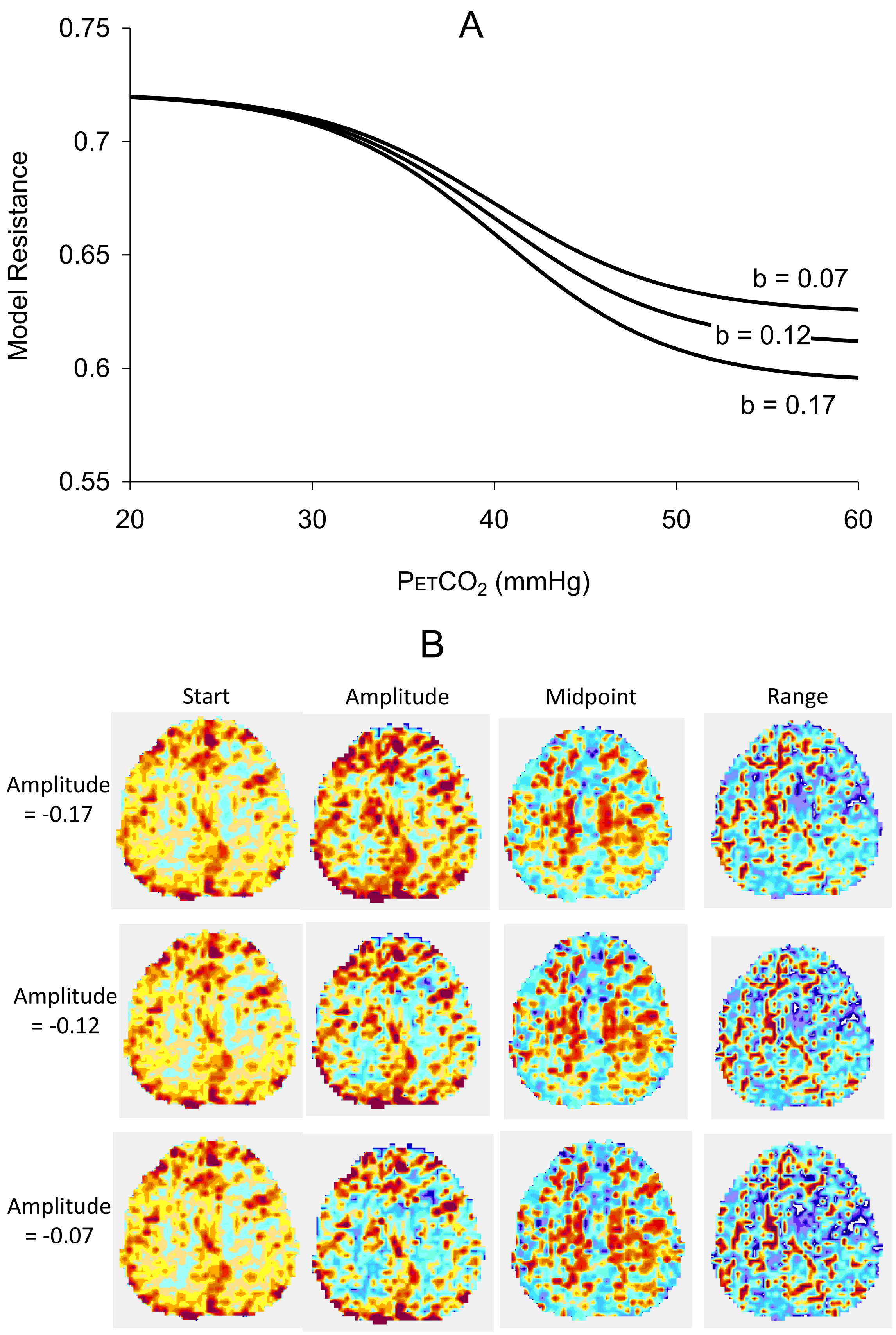
A
0.75
0.7
0.65
0.6
0.55
20
30
40
50
60
Model Resistance
PETCO2 (mmHg)
b = 0.07
b = 0.12
b = 0.17
B
Start
Amplitude
Midpoint
Range
Amplitude = -0.17
Amplitude = -0.12
Amplitude = -0.07

Figure 4: The effects of a change in reference resistance sigmoid parameter b (Amplitude) on model resistance (A) and an example of examined voxels resistance sigmoid parameter maps (B).

Reference resistance parameters (Ranget)

Reference resistance sigmoid parameter d (Range) variation changes the shape of the model resistance sigmoids (Figure 5A) without substantially affecting the examined voxels resistance sigmoid parameter maps (Figure 5B).

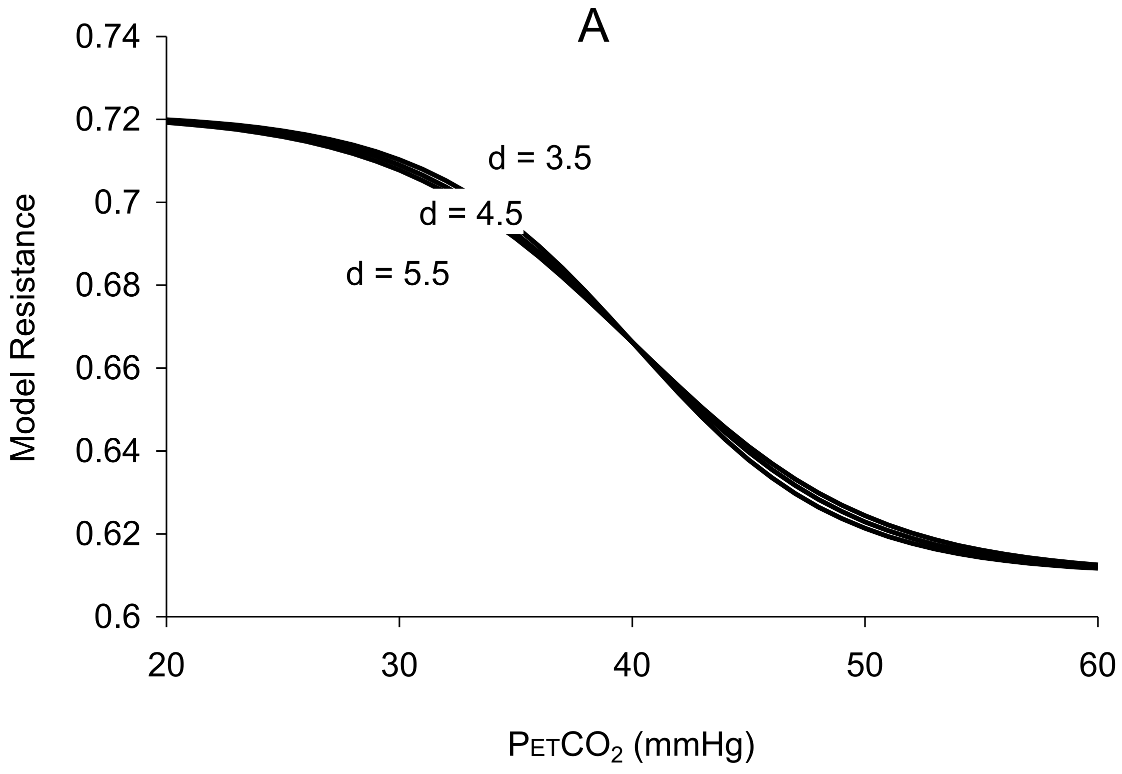

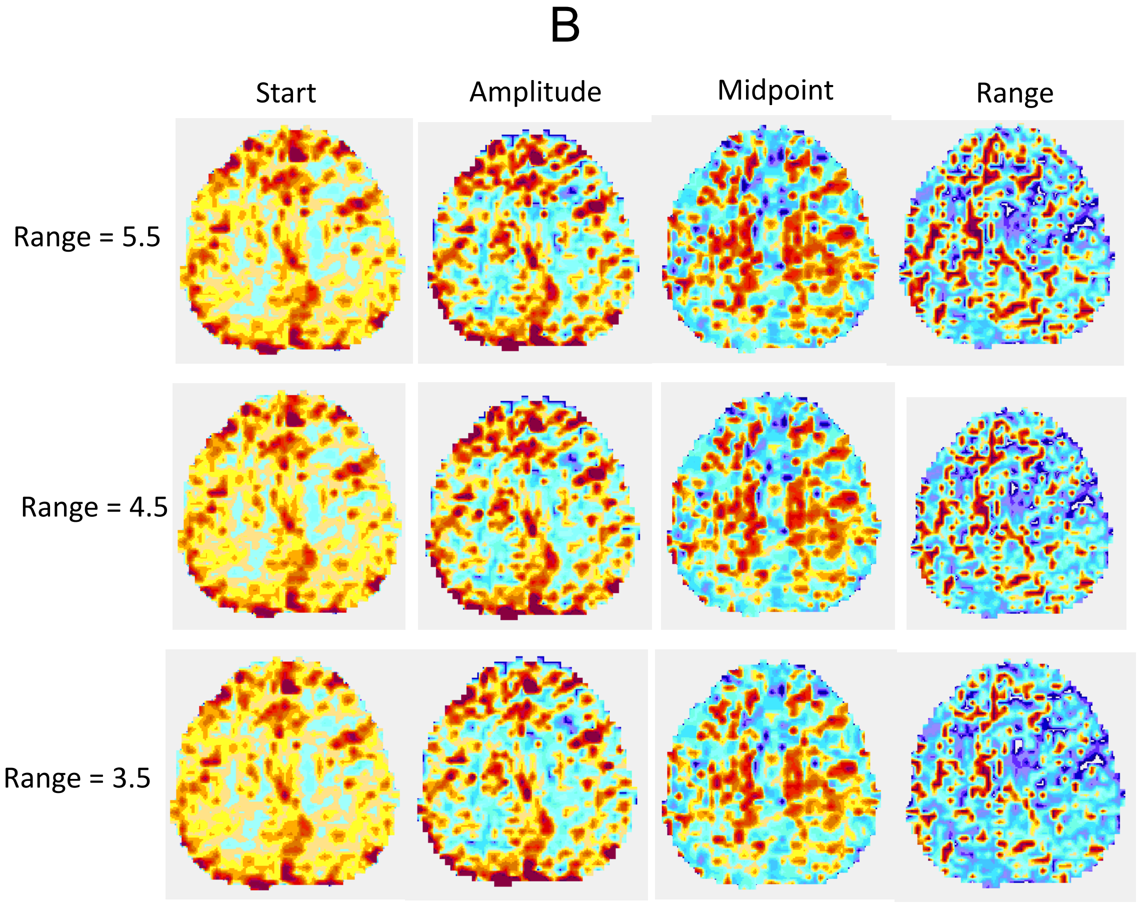

Figure 5: The effects of a change in reference resistance sigmoid parameter d (Range) on model resistance (A) and an example of examined voxels resistance sigmoid parameter maps (B).

Reference resistance parameters (Midpoint)

The reference resistance sigmoid parameter c (Midpoint) is of major importance since it designates the $\mathrm{P_{ET}CO_2}$ at which the reference resistance responsiveness to CO_2 is maximum. The voxel resistance sigmoid midpoint parameter therefore determines the $\mathrm{P_{ET}CO_2}$ range over which the voxel resistance is effective; a lower midpoint exposes that voxel to steal in the hypercapnic range (type B +/-), whereas a higher midpoint exposes the voxel to steal in the hypocapnic range (type D -/+) of the ramp CO_2 challenge.

If the resistance regulation ability of healthy regions of the brain is to be maximum at the resting $\mathrm{P_{ET}CO_2}$, then the resistance sigmoid midpoint should be the same as resting $\mathrm{P_{ET}CO_2}$. This correspondence appears to be the case. As Figure 6 shows, resting $\mathrm{P_{ET}CO_2}$ correlates with the average midpoint of healthy regions in both controls and patients (Pearson correlation coefficient 0.613 $P<0.001$).

.

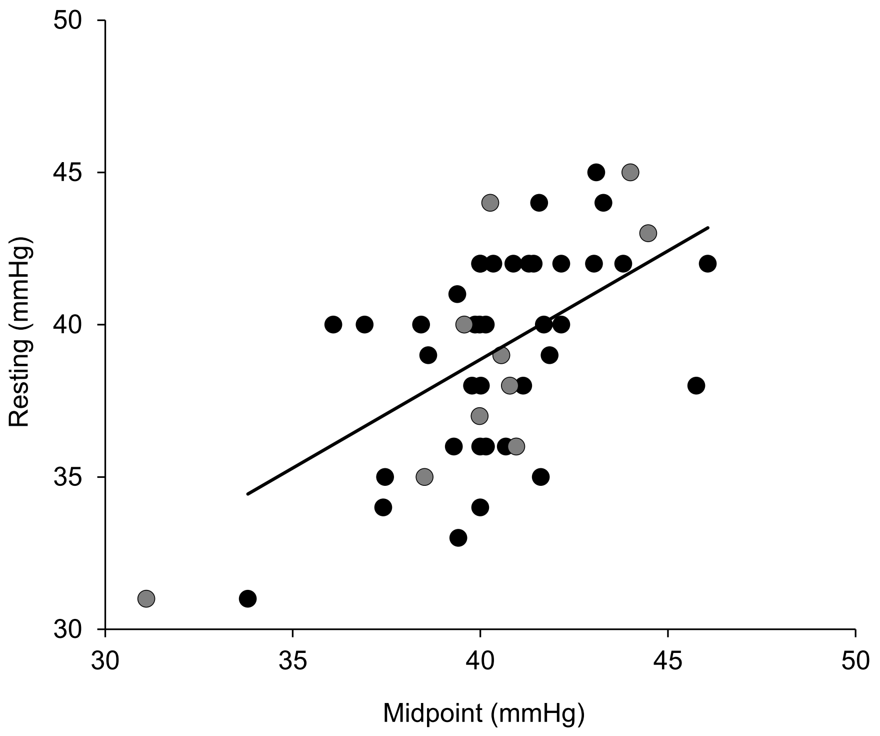

Figure 6: Resting $P_{ET}CO_2$ (mmHg) vs. the midpoint $P_{ET}CO_2$ (mmHg) found for regions with a robust CVR in 38 control subjects (black circles) and 10 patients (gray circles) with steno-occlusive disease.

In our survey of control subjects, the modal midpoint found for regions with a robust CVR (reported in Figure 8 in the main text) was 40 mmHg and this value was chosen as the reference resistance sigmoid midpoint. It might be argued that the individual subject's midpoint should be the reference midpoint but then the reference resistance sigmoid would not be standardised between subjects. We chose to make this standardisation, with the consequence that examined voxels resistance sigmoid midpoint parameter maps could be biased. The relative differences in midpoint between regions should nevertheless be preserved. Figure 7 illustrates this point; adjustments of midpoint map scaling restore a similar appearance despite the major changes in reference resistance sigmoid parameter from 30 to 40 to 50 mmHg. It should be noted that the range maps are altered as well (Figure 7).

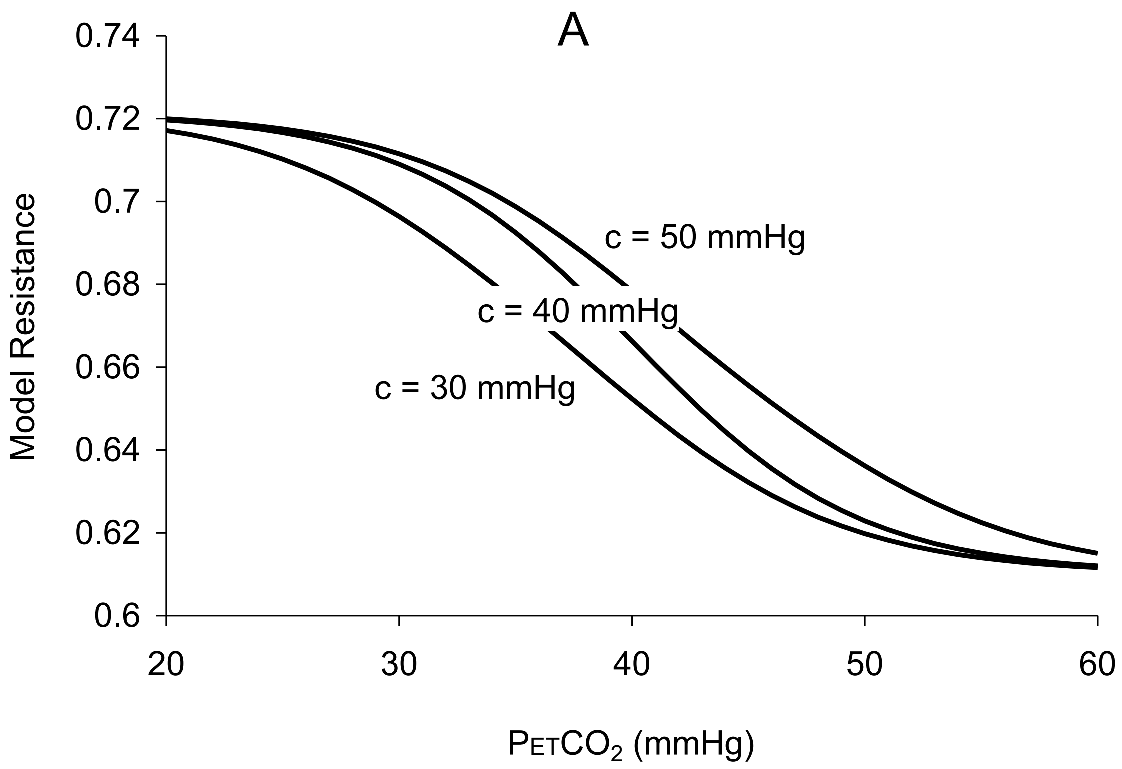
A
0.74
0.72
0.7
0.68
0.66
0.64
0.62
0.6
Model Resistance
c = 50 mmHg
c = 40 mmHg
c = 30 mmHg
20
30
40
50
60
PETCO2 (mmHg)

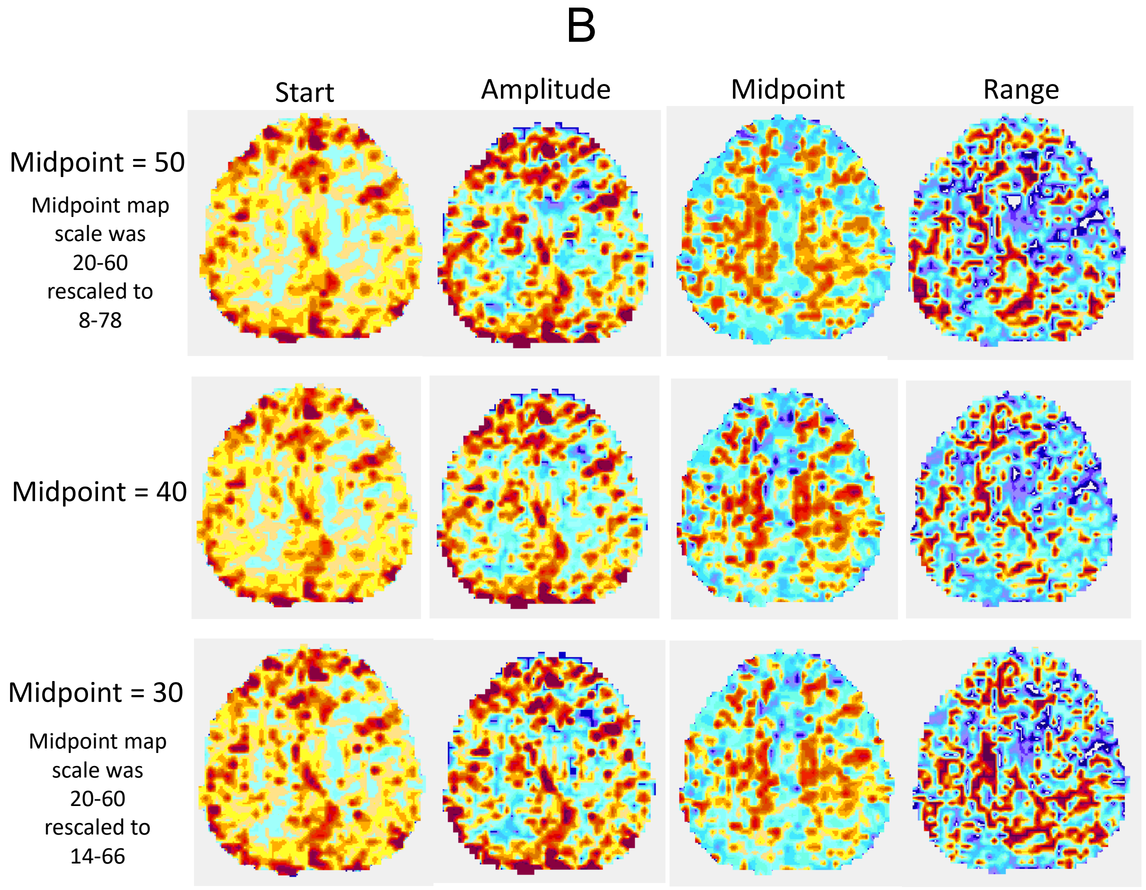
B
Start
Amplitude
Midpoint
Range
Midpoint = 50
Midpoint map scale was 20-60 rescaled to 8-78
Midpoint = 40
Midpoint = 30
Midpoint map scale was 20-60 rescaled to 14-66

Figure 7: The effects of a change in reference resistance sigmoid parameter c (Midpoint) on model resistance (A) and an example of examined voxels resistance sigmoid parameter maps (B).

Support for our choice of reference resistance sigmoid midpoint was also drawn from the control subjects as well as from an examination of 10 patients with steno-occlusive disease. As the histogram below shows, while the resting $P_{ET}CO_2$ were quite varied, the resistance sigmoid midpoints for regions with a robust CVR tended to be concentrated near 40 mmHg. We suggest that a midpoint $P_{ET}CO_2$ of 40 mmHg corresponds with the preferred PCO_2 for healthy brain tissue.

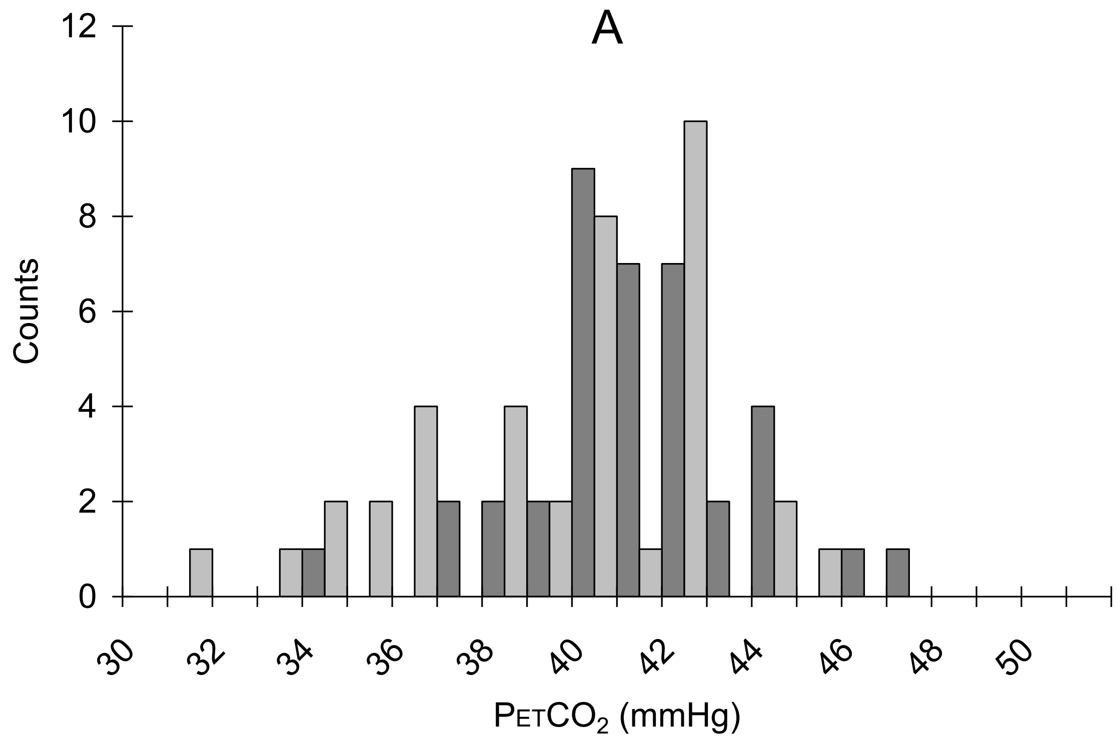

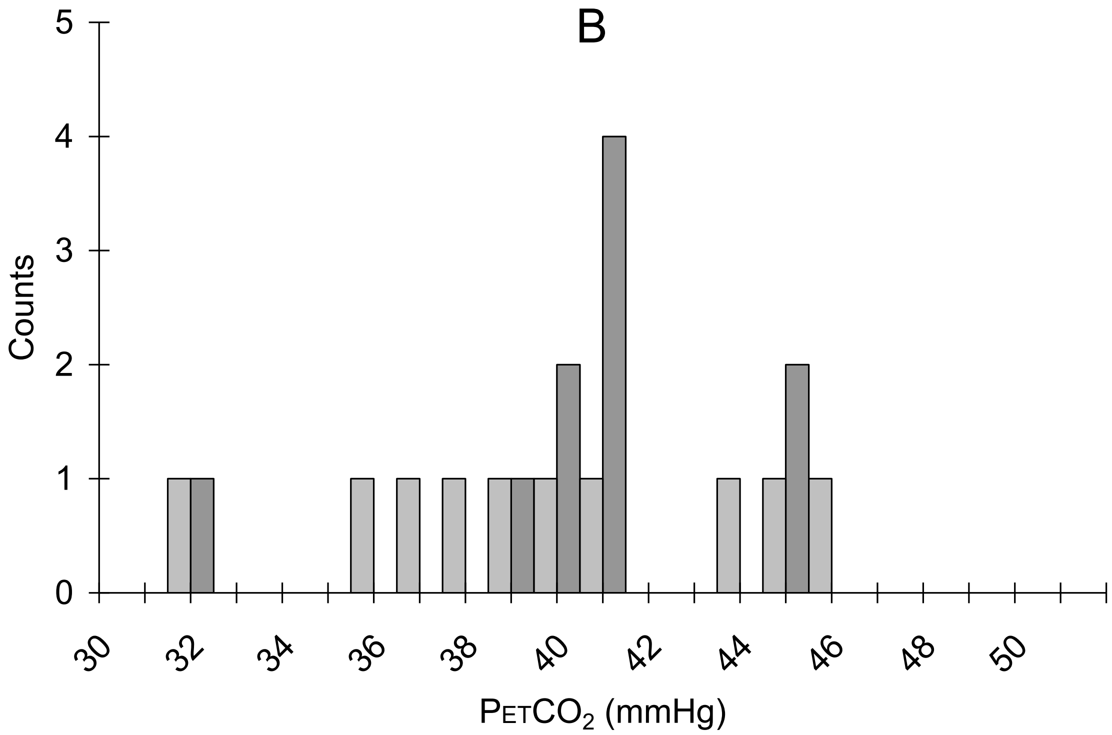

Figure 8: A histogram showing the distribution of resistance sigmoid midpoints for regions with a robust CVR (dark gray) and resting $P_{ET}CO_2$ (light gray). A) Control subjects. B) Patients with steno-occlusive disease.

Finally, for the control subject (Figure 7), histograms of the distributions of examined voxel resistance sigmoid midpoints for all voxels (Figure 9) are similar despite differences in the midpoint parameter of the reference resistance sigmoid (30, 40 and 50 mmHg). This finding also supports the choice of a fixed reference resistance sigmoid midpoint.

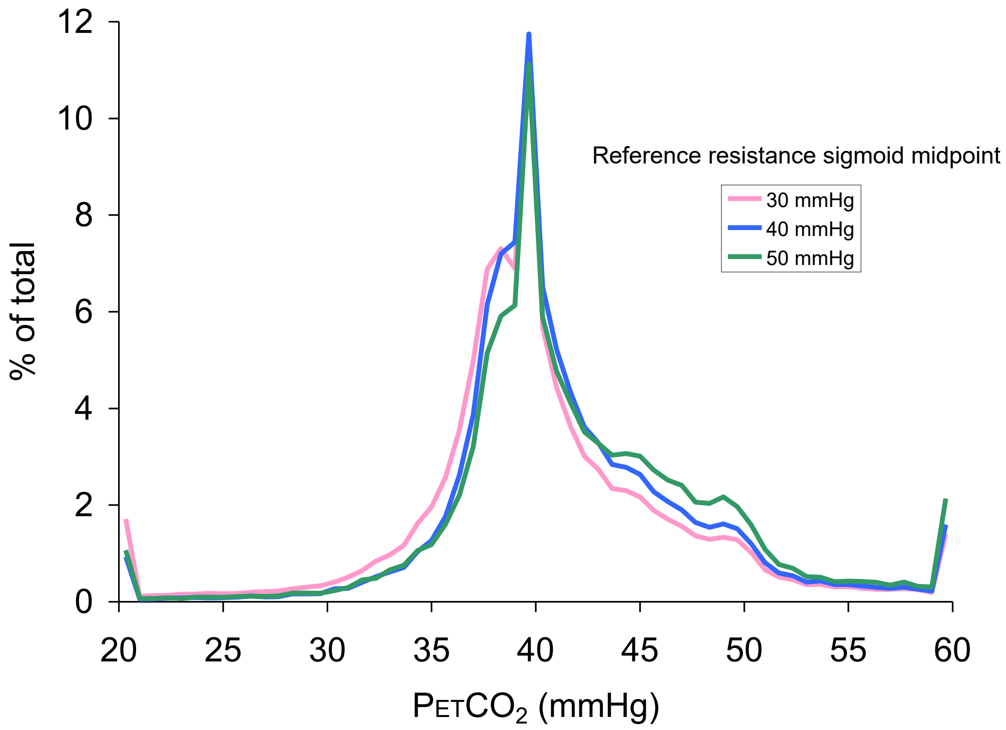

Figure 9: Histograms of the distributions of examined voxel resistance sigmoid midpoints for all voxels for reference resistance sigmoid midpoints of 30, 40 and 50 mmHg.

Resistance model of vasodilatory reserve deficit volume compared to surrounding healthy brain

In consideration of the limitations to the use of a "standard" competition model, instead of considering one voxel at a time, consider a simple model of competing brain regions (Figure 10). For simplicity, first consider totally healthy brain where the distal resistance in hypercapnia is half its value at normocapnia. We introduce a small volume with vasodilatory reserve deficit (where no change in resistance occurs with hypercapnia), such that the pressure head proximal to the distal resistance at normocapnia is unaffected by the deficit. This condition arises because the overall resistance of the huge set of parallel resistances in the surrounding healthy brain is much lower than the resistance of any small volume so the effective total parallel resistance controlling the pressure is completely dominated by the large volume of healthy brain.

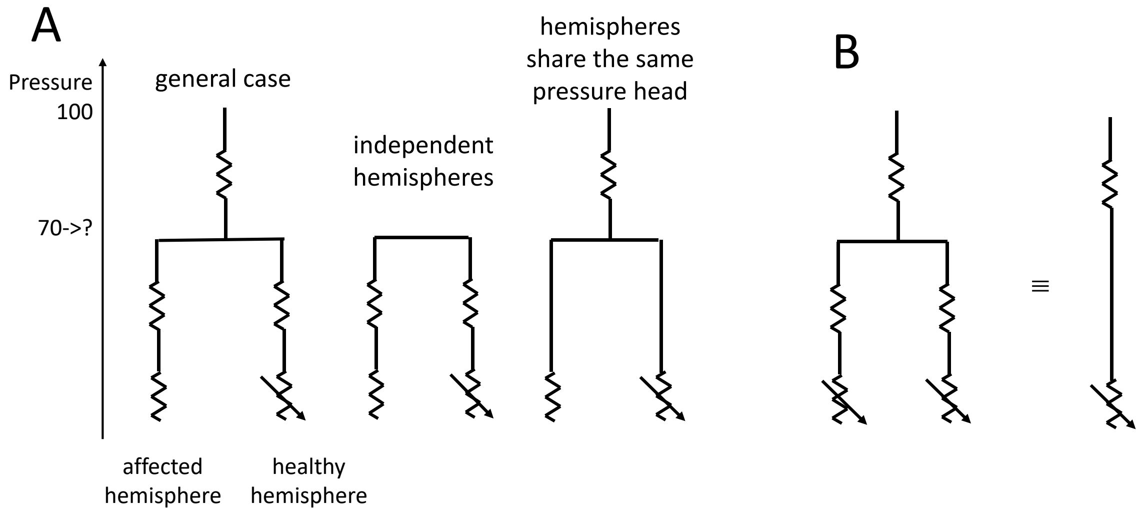

Figure 10: A model of competing brain regions A) One hemisphere with zero vasodilatory reserve; other hemisphere healthy. B) Small region with zero vasodilatory reserve; pressure controlled by overall healthy hemispheres

Arterial resistances were calculated to provide pressure head of 70% MAP at normocapnia for extreme situations (independent and shared pressure cases), and the change in pressure was calculated for healthy distal resistances in hypercapnia equal to half their normocapnic value. Affected regions assumed to have no change in resistance at hypercapnia, so % change in flow for these regions = % change in pressure. Given a 30% pressure drop across the proximal arteries in normocapnia, this situation results in a 23% drop in flow (a steal effect) in the vasodilatory reserve deficit volume, with a 54% increase in flow in the healthy volume (Table 1). As we increase the deficit volume, the above result holds as long as the total healthy distal resistance in normocapnia remains sufficiently small compared to the total deficit distal resistance. In summary, this means that the deficit volume should remain reasonably small compared to the total of both hemispheres. In this regime, a “standard” competition model is valid.

Table 1: Comparison of calculated steal effect on a) one affected hemisphere compared to healthy other hemisphere, b) small region of CVR deficit

	% $\Delta P_{affected}$	% $\Delta F_{affected}$	% $\Delta F_{healthy}$
a) Hemisphere deficit			
Independent hemispheres	0	0	+54%
Hemispheres share pressure head	-13%	-13%	+74%
b) Small region deficit	-23%	-23%	+54%

Since the deficit can be ignored here in terms of the effect on pressure just proximal to the distal resistances, the resistance model is symmetrical between

hemispheres. This attribute means that the model needed to predict the relevant pressure can be derived in terms of a simple non-branching model (see Figure 10). When the vasodilatory reserve deficit volume becomes too large for the assumption above to hold (i.e. the deficit now has a significant influence on its proximal pressure head), then the hemispheric symmetry assumption also fails and we have to consider some details concerning the arterial resistances proximal and distal to the point where the arteries branch to each hemisphere (see Figure 10). There are two ends to this range: at one extreme, the distal resistances in each hemisphere share the same pressure head; at the other extreme the two hemispheres have independent pressure heads.

Consider a vasodilatory reserve deficit volume equal to one hemisphere. If the hemispheres act independently, there is no steal (from surrounding brain) resulting in 0% flow change (no steal). If the hemispheres share the same pressure head, there will be a 13% drop in flow (steal) in the deficit hemisphere and a 74% increase in flow in the healthy hemisphere. Overall, we may expect a range in steal from 13% to 23% in going from equal vasodilatory reserve deficit and healthy volumes to a smaller proportion of vasodilatory reserve deficit volume. If the "healthy" brain is actually somewhat compromised (e.g. distal resistances drop to ¾ of NC value instead of ½) this will of course reduce the steal.

We conclude that most of these limitations to the use of a "standard" competition model arguably affect between subject comparisons rather than within subject maps.
